# The experience of UK patients with bladder cancer during the second wave of the COVID‐19 pandemic

**DOI:** 10.1002/bco2.153

**Published:** 2022-05-17

**Authors:** Beth Russell, Sarah Spencer‐Bowdage, Jeannie Rigby, Jackie O'Kelly, Phil Kelly, Mark Page, Caroline Raw, Paula Allchorne, Peter Harper, Jeremy Crew, Roger Kockelbergh, Allen Knight, Mieke Van Hemelrijck, Richard T. Bryan

**Affiliations:** ^1^ Translational Oncology and Urology Research King's College London London UK; ^2^ Action Bladder Cancer UK Tetbury UK; ^3^ Barts Health NHS Trust London UK; ^4^ Leaders in Oncology Care London UK; ^5^ Oxford University Hospitals NHS Foundation Trust Oxford UK; ^6^ University Hospitals of Leicester NHS Trust Leicester UK; ^7^ Leicester Cancer Research Centre University of Leicester Leicester UK; ^8^ World Bladder Cancer Patient Coalition Brussels Belgium; ^9^ Bladder Cancer Research Centre, Institute of Cancer and Genomic Sciences University of Birmingham Birmingham UK

**Keywords:** bladder cancer, COVID‐19, patient experience, survey

The first wave of the COVID‐19 pandemic placed unprecedented strains on healthcare systems worldwide, with significant detriment to the routine diagnosis and follow‐up of non‐COVID patients. For UK bladder cancer (BC) patients, our previous survey captured and reported some of these detriments.^1^ By the arrival of the second wave (peaking in January 2021 and ending in April 2021),^2^ the UK's NHS had adjusted clinical pathways in line with national and international guidelines in order to reduce such disruption. Through this second survey, we investigated whether such reconfiguration had mitigated the impact of the second wave on BC patients.

The survey was distributed via the SurveyMonkey platform (San Mateo, CA, USA) from 26 January to 30 May 2021. As previously, patients were directed to this survey via the Action Bladder Cancer (ABC) UK website (http://actionbladdercanceruk.org/), ABC UK Patient Support Groups and social media platforms. Anonymized demographic and tumour‐specific characteristics were collected. Analysis of these data was approved by the King's College London Research Ethics Office. Data were predominantly analysed descriptively; chi‐squared and Fisher's exact tests were conducted to compare changes to treatment and monitoring due to COVID‐19 between age groups, sex and diagnosis (NMIBC/MIBC). Kruskal–Wallis tests were undertaken to determine differences in the answers to the questions ‘How concerned do you feel about COVID’ and ‘How concerned do you feel about COVID‐19 affecting your BC treatment and monitoring?’ between age groups, sex, diagnosis and treatment groups. All analyses were conducted using STATA/MP 17.0 (Texas, USA).

A total of 134 participants consented to take part in the survey (versus 156 in the first survey); here, we present results from the 95 participants who answered the questions (vs. 156 patients in the first survey). Fewer than one‐quarter (23%) of respondents reported participation in our first survey in 2020. Most respondents lived in England (90%), were over the age of 60 (70%) and were male (57%); a larger proportion of female respondents answered this survey compared with the first (43% vs. 28%) (Table S1).

Regarding BC diagnosis, 75% reported having NMIBC, 15% with MIBC and 3% with advanced disease, and 7% were unsure of their stage (Table S1). The majority of respondents (64%) were undergoing monitoring rather than active treatment, 29% were on active treatment, and a small proportion had been discharged from follow‐up (6%).

When asked to reflect upon their treatment during 2020, the majority of respondents reported that their treatment was delivered when expected (67%), whilst 75% of respondents reported that their BC monitoring appointment occurred when expected (Figure 1). Fourteen patients responded that their treatments were either cancelled or delayed. Fisher's exact tests did not reveal significant differences in treatment disruption between age groups, sex and NMIBC/MIBC. However, for patients undergoing monitoring/follow‐up, statistically significant differences were observed for age groups (*p* = 0.020) and NMIBC/MIBC (*p* = 0.006)—a higher proportion of younger patients were not scheduled for monitoring (older patients mostly reported that their monitoring occurred as expected), and more patients with NMIBC had their monitoring appointment postponed or cancelled (*n* = 17) compared with MIBC patients (*n* = 1).

**FIGURE 1:**
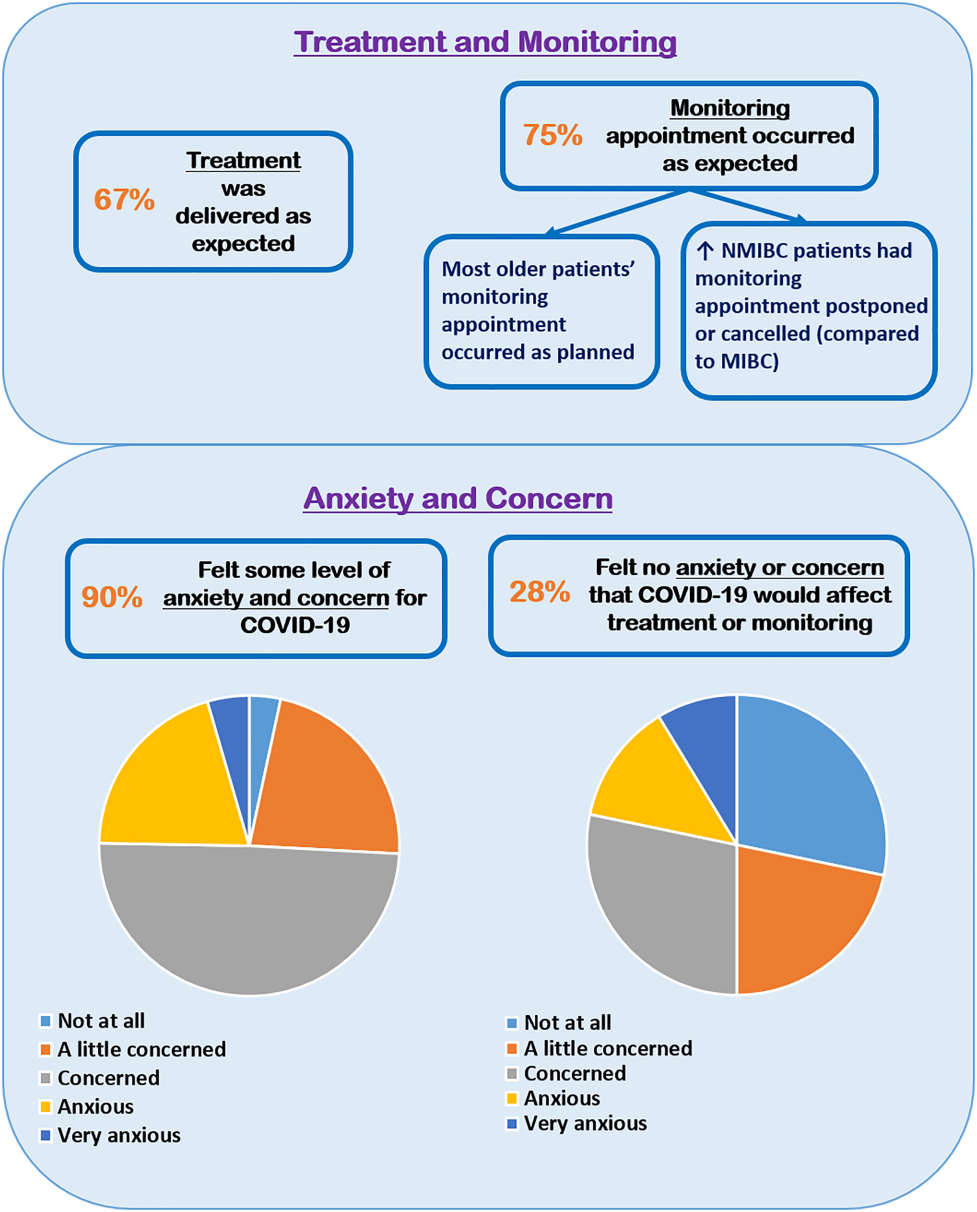
Overview of key findings. MIBC, muscle invasive bladder cancer; NMIBC, non‐muscle invasive bladder cancer

Almost all respondents reported feeling some level of anxiety or concern about COVID‐19 (90%) (Figure 1).

Conversely, regarding concerns about COVID‐19 affecting BC treatment or monitoring, one‐third of respondents (28%) felt no concern at all (similar to Survey 1, 26%), 9% reported feeling very anxious, and the majority of patients reported feeling a little concerned or concerned (50%) (Figure 1). Again, these were similar to the first survey in which 51% reported feeling a little anxious and 22% very anxious.

Over 60% of respondents reported feeling as supported or more supported during the pandemic than before the pandemic. When participants were asked to comment on the changes made by their hospital in response to the pandemic, seven patients reported that the implemented changes were positive—the efficiency of telephone appointments and use of different hospitals or buildings were specifically highlighted as positives. Notwithstanding, participants reported negative experiences of visiting hospital appointments alone; for example, one respondent wrote, ‘Not being able to bring anybody with you to consultations as I suffer from MCI (mild cognitive impairment)’.

Our first survey, conducted during and immediately after the first wave of the pandemic in the United Kingdom, reported that 49% of patients experienced disruption to their treatment or follow‐up (delays, postponements or cancellations/curtailments).^1^ This survey appears to report the resumption of a more ‘normal’ service within the following 9 months, with over two‐thirds of patients reporting that their treatment and monitoring had occurred as expected. Notably, considerably fewer patients responded to this survey than the previous survey, suggesting an overall ‘satisfaction’ with the care received.^3^, ^4^ These data demonstrate an impressive turnaround in the delivery of care to BC patients, despite the substantial incumbent challenges of the UK's second wave of the pandemic. This is testimony to the resilience and determination of the NHS and its healthcare professionals, a resilience and determination that will be tested again as both the Omicron and Deltacron variants propagate in the United Kingdom and worldwide. Anecdotally, a number of urology units continue to experience considerable disruption, indicated here by a not insubstantial minority of respondents still describing delays to treatment and monitoring.

Here, we report that most patients did not experience a delay to their treatment or monitoring appointments during the study period. Other studies have also investigated the effect of the pandemic on the time to BC treatment. Ferro et al investigated the impact of the pandemic on time to primary and secondary resection and adjuvant intravesical therapy in high‐risk NMIBC patients^5^: Although no significant differences in TURBT quality were identified, a delay in treatment schedule and disease management was observed. The authors highlighted the need to investigate the oncological impacts of these delays,^5^ as we did in our first report.^1^

A strength of this study is that the patients who responded to this survey are representative of the general BC population in their distribution of age, sex and bladder stage grouping (NMIBC and MIBC). However, the sample size of 95 respondents represents only a very small minority of the UK's BC patient community.

Despite many positive changes (including the use of telephone consultations and alternative infrastructure) and that half of respondents had been vaccinated at the time of the survey, most patients understandably still feel anxiety and concern for COVID‐19 and the impact the pandemic has had, or may have, on their BC care. With the pandemic relentlessly continuing into 2022, it is imperative that research also continues to monitor the impact on BC patients such that longer‐term planning for the inevitable detriments can be well informed.

## CONFLICT OF INTEREST

R. T. Bryan has contributed to advisory boards for Olympus Medical Systems and Janssen and undertakes research funded by UroGen Pharma and QED Therapeutics. ABC UK is a UK‐registered charity and receives income from a variety of sources including public donations and grants, as well as educational grants from corporate supporters. In 2020 to date, ABC UK has received educational grants from Bristol Myers Squibb, Janssen‐Cilag Ltd, Merck Serono Limited and Pfizer UK.

## Supporting information




**Table S1.** Characteristics of survey respondents.
