# Supplementary material for: The experience of UK patients with bladder cancer during the second wave of the COVID‐19 pandemic
Source: BJUI Compass. 2022 May 17;3(5):324–6. doi: 10.1002/bco2.153 (PMC9348423; doi:10.1002/bco2.153)
Supplement: Supplementary file 1 — Table S1. Characteristics of survey respondents. [file BCO2-3-324-s001.docx]

**Supplementary Table 1. Characteristics of survey respondents.**

| **Cohort Characteristics** | | |
| --- | --- | --- |
|  | **N**  **(Total N=95)** | **%** |
| **Age Group** |  |  |
| 30-49 | 8 | 8.40 |
| 50-59 | 20 | 21.10 |
| 60-69 | 35 | 36.80 |
| 70-79 | 25 | 26.30 |
| 80 and older | 7 | 7.40 |
| **Sex** |  |  |
| Female | 40 | 42.10 |
| Male | 55 | 57.90 |
| **Diagnosis** |  |  |
| NMIBC | 71 | 74.70 |
| MIBC | 17 | 17.90 |
| Unknown | 7 | 7.40 |
| **Treatment paradigm** |  |  |
| Active Treatment | 28 | 29.50 |
| Monitoring | 61 | 64.20 |
| Discharged | 6 | 6.30 |
| **Active Treatment Type (n=28)** |  |  |
| Initial TURBT | 3 | 10.70 |
| A course/courses of mitomycin | 4 | 14.30 |
| A course/courses of BCG | 14 | 50.00 |
| Neoadjuvant chemotherapy | 1 | 3.60 |
| Radiotherapy | 2 | 7.10 |
| Cystectomy (planned or completed) | 1 | 3.60 |
| Newly diagnosed, awaiting to start treatment | 2 | 7.10 |
| Unknown | 2 | 7.10 |

*NMIBC – Non-muscle invasive bladder cancer; MIBC – muscle invasive bladder cancer; TURBT – transurethral resection of the bladder tumour; BCG - Bacillus Calmette–Guérin.*
